# Non-beta-lactam agents for definitive treatment of ampicillin-susceptible *Enterococcus* bacteremia: a single-center experience

**DOI:** 10.1017/ash.2025.10078

**Published:** 2025-08-11

**Authors:** HeeEun Kang, Asif N. Khan, Justin J. Kim, Isabella W. Martin, Richard A. Zuckerman

**Affiliations:** 1 Infectious Disease and International Health, Dartmouth-Hitchcock Medical Center, Lebanon, NH, USA; 2 Geisel School of Medicine at Dartmouth College, Hanover, NH, USA; 3 Pathology and Laboratory Medicine, Dartmouth-Hitchcock Medical Center, Lebanon, NH, USA

## Abstract

**Objective:**

To describe the use of non-beta-lactam agents (NBL) to treat ampicillin-susceptible *Enterococcus* bacteremia (ASEB), and to identify factors associated with their use.

**Methods:**

A single-center retrospective study at a rural tertiary referral center was conducted to identify ASEB episodes between January 1, 2016, and 31 December, 2021. Patient, microbiological, infection, clinical management characteristics, and outcomes were compared between those who received NBL versus BL agents for definitive therapy. Multivariable logistic regression analysis was used to determine factors associated with NBL use.

**Results:**

158 episodes of ASEB in 153 patients were included. 43 episodes (27%) were treated with NBL for definitive therapy. Factors associated with NBL therapy were younger age, history of penicillin allergy, history of cancer, end-stage renal disease (ESRD), polymicrobial bacteremia, lack of metastatic foci, and lack of endocarditis. Combination therapy was used in 23% of those treated with BL therapy versus zero patients receiving NBL therapy. All-cause 30-day and 90-day mortality and 30-day relapse rate were not statistically different. In the regression model, NBL therapy was more likely in those with: younger age (AOR 0.95, *p* < .01), any penicillin allergy (AOR 5.87, *p* < .01), history of cancer (AOR 5.25, *p* < .01), ESRD (AOR 12.48, *p* < .001), and polymicrobial bacteremia (AOR 4.20, *p* < .01).

**Conclusion:**

NBL was used as definitive treatment in 27% of ASEB with good clinical outcomes. This real-life experience suggests NBL can be successfully used to treat ASEB based on clinical discretion.

## Introduction

Enterococci are Gram-positive, facultative anaerobic bacteria that colonize the human gastrointestinal and female genitourinary tract. Enterococci are hardy organisms – capable of withstanding extreme temperatures, disinfectants, and high osmolality^1^ – that are increasingly recognized as an important cause of nosocomial infections.^2,3^ *Enterococcus* bacteremia (EB) is associated with significant mortality and morbidity. The average 30-day mortality among patients who develop EB ranges from 20–35%,^4^ and the average hospital length of stay is 14–38 days.^5,6^ EBs are expensive to treat: one study estimated that the average healthcare cost associated with EB was $41,233 in 2022.^6^

Historically, beta lactam agents (BL) such as ampicillin and piperacillin-tazobactam were used to treat ampicillin-susceptible *Enterococcus* bacteremia (ASEB). Since the development of non-beta lactam agents (NBL) with activity against *Enterococcus* such as vancomycin, daptomycin, and linezolid, these newer agents are also being used to treat ASEB. There are many studies highlighting the use of NBL for infections caused by vancomycin-resistant *Enterococcus* (VRE). Published research on NBL use for treatment of ASEB has focused on vancomycin, and shows mixed outcomes.^7–9^ It is unclear how often clinicians reach for NBL to treat ASEB, what patient or infection characteristics are associated with treatment with NBL instead of BL, and if NBL agents such as daptomycin are as efficacious and safe as vancomycin or BL agents in treating ASEB.

The objectives of this study were: (1) to determine the rate of NBL use for definitive treatment of ASEB, (2) to identify factors associated with NBL use for definitive treatment of ASEB, and (3) to evaluate the clinical outcomes of 30- and 90-day all-cause mortality and relapse rates in patients with ASEB who receive NBL vs BL therapy.

## Methods

This is a single-center, retrospective cohort study conducted at Dartmouth Hitchcock Medical Center, a rural tertiary care academic medical center in New Hampshire, USA. We obtained a list of all positive blood cultures with *Enterococcus* spp. growth from the clinical microbiology laboratory during the six-year study period (1/1/2016–12/31/2021). Rapid molecular testing for positive blood culture specimens using Blood Culture Identification (BCID, and later BCID2; BioMérieux, Durham, NC) was in use during the entire study period, which allowed for early identification of VRE by detection of *vanA*/*vanB* gene. Inclusion criteria for the study were: (1) one or more positive *Enterococcus* spp. blood culture specimen, (2) age 18 years or older, and (3) bacteremia treated as inpatient. Exclusion criteria were: (1) provider determination that the positive blood culture was not a true infection, (2) death or transition to hospice or comfort care before antibacterial regimen was finalized for definitive therapy by the treating provider, (3) same episode bacteremia, defined as multiple positive blood cultures with the same *Enterococcus* species within a seven-day period. A patient could have multiple bacteremia episodes if the bacteremia were separated by greater than seven days with interval negative blood cultures.

For each bacteremia episode, we collected data on patient characteristics including severity of illness as determined by a list of author-defined preexisting conditions and Charlson Comorbidity Index (CCI). The author-defined preexisting conditions list was made using the comorbidities reported in the published literature describing enterococcal bacteremia and included liver foreign body, underlying gastrointestinal or genitourinary disease, and other conditions (Supplementary Appendix Table 1). We obtained data on clinical microbiology results (species and antimicrobial susceptibility testing result), infection characteristics (duration of bacteremia, source, metastatic foci including endocarditis), clinical management (antibiotic selected for definitive therapy including combination therapy, Infectious Disease [ID] consultation, echocardiogram), and clinical outcome (all-cause 30- and 90-day mortality, 30-day relapse, and hospital length of stay). For daptomycin, susceptible dose-dependent (SDD; new category for *E. faecium* described in the Clinical and Laboratory Standards Institute breakpoint revision in 2019) was classified as susceptible for the analysis. Definitive therapy was defined as the antibiotic(s) chosen at the time of finalization of antibiotic plan by the treating physician, after work-up to determine the source, metastatic sites of infection, and attempts at source control when applicable. Combination therapy was defined as the addition of ceftriaxone or gentamicin to BL or NBL to treat endocarditis or other serious manifestations of enterococcal infection, and not the addition of other antimicrobials to treat concurrent non-enterococcal infections. The primary outcome was the type of antimicrobial agent used for definitive therapy (NBL vs BL). Secondary outcomes were all-cause 30- and 90-day mortality, 30-day relapse, and length of stay.

Data was collected via Cerner Millenium (for the initial list of patients), the Dartmouth Health Analytics Institute, and manual chart review (performed by HK and ANK) and entered into RedCap^10,11^ hosted at Dartmouth-Hitchcock Medical Center. Statistical analyses were performed using R (version 4.3.2).^12^ Statistical significance was set at *P* < .05. To test for the null hypothesis, chi-squared and Fisher’s exact test were used for categorical variables, and t-test was used for continuous variables. Logistic regression was used for multivariable analysis using the variables identified to be statistically significant from initial analysis, excluding serious penicillin allergy and metastatic infection as these were felt to be substantially similar (colinear) with any penicillin allergy and endocarditis. Multicollinearity was checked using variance inflation factor (VIF). The study protocol was reviewed and approved by Dartmouth Health Institutional Review Board.

## Results

**Figure 1.**
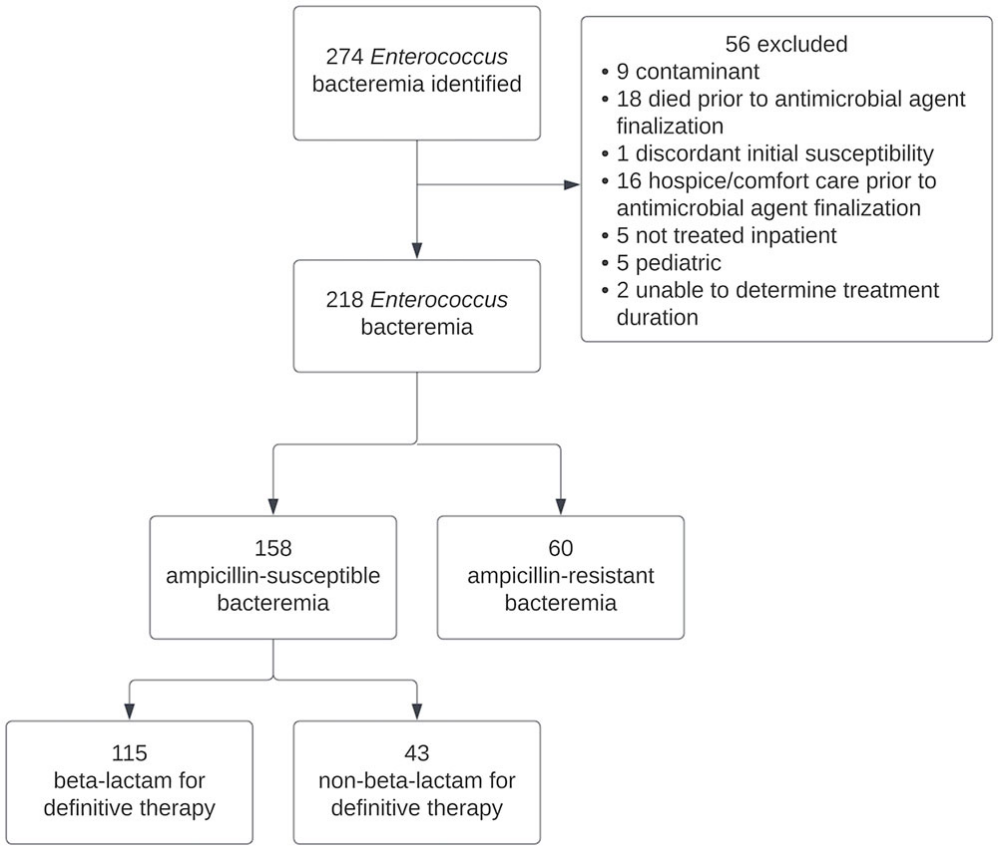
CONSORT flow diagram of patients included in study.

A total of 158 episodes of ASEB in 153 patients were included in this analysis. 43 episodes (27%) were treated with NBL for definitive therapy (Figure 1). The proportion of patients treated with NBL did not change significantly throughout the study period (Supplementary Appendix Table 2). The most commonly used NBLs for definitive therapy were: vancomycin (29%), linezolid/tedizolid (19%), and daptomycin (16%). The most commonly used BLs for definitive therapy were: ampicillin (57%), piperacillin-tazobactam (15%), and amoxicillin-clavulanate (11%) (Table 1).

**Table 1.** Antibiotics used to treat ampicillin-susceptible *Enterococcus* bacteremia for definitive therapy

	Beta lactam (*n* = 115)		Non-beta lactam (*n* = 43)	
	Amoxicillin	12 (10%)	Dalbavancin	2 (5%)
	Amoxicillin-clavulanate	13 (11%)	Daptomycin	7 (16%)
	Ampicillin	65 (57%)	Fluoroquinolone	5 (12%)
	Ampicillin-sulbactam	3 (3%)	Linezolid/Tedizolid	8 (19%)
	Ertapenem	1 (1%)	Vancomycin	21 (29%)
	Penicillin	4 (3%)		
	Piperacillin-tazobactam	17 (15%)		
Combination therapy	Ceftriaxone	26 (23%)		0 (0%)

The majority of patients were male, White, and neither Hispanic nor Latino. The patients had a high degree of morbidity as noted by the high CCI, with no difference between the NBL and BL groups. The vast majority (98%) of patients had at least one of the 18 author-defined preexisting conditions, with the average being over two. Factors associated with NBL use were: younger age (58 vs 66 years old, *P* = .02), any penicillin allergy (42% vs 9%, *P* < .01), serious penicillin allergy (14% vs 1%, *P* < .01), history of cancer (37% vs 17%, *P* = .01), and end-stage renal disease (ESRD) (23% vs 3%, *P* < .01).

Microbiologically, *E. faecalis* was the most common causative organism causing ASEB (84%), followed by *E. faecium* (12%). Daptomycin resistance was more common among those receiving NBL (eg, linezolid/tedizolid, fluoroquinolones) vs BL (7% vs 0%, *P* = .02). Other antimicrobial resistance or species were not significantly associated with NBL therapy.

Of the infection characteristics studied, polymicrobial bacteremia (40% vs 16%, *P* < .01), lack of metastatic foci of infection (12% vs 31%, *P* = .02), and lack of endocarditis (7 vs 23%, *P* = .02) were significantly associated with NBL therapy.

Combination therapy was used in 23% of those treated with BL compared to no patients receiving NBL (*P* < .01). There was no difference between all-cause 30-day mortality, all-cause 90-day mortality, or 30-day relapse rate between the two groups (Table 2).

**Table 2.** Baseline characteristics of patients treated with non-beta lactam versus beta lactam agents for definitive therapy for ampicillin-susceptible *Enterococcus* bacteremia

	Non-beta lactam *N* = 43	Beta lactam *N* = 115	*P* value
Patient			
Age (year) (IQR)	58 (43, 74)	66 (55, 77)	**.02**
Sex: Male	27 (63%)	81 (70%)	.47
Race: White	42 (98%)	112 (97%)	1
Ethnicity: Not Hispanic or Latino	43 (100%)	113 (98%)	1
Penicillin allergy, any	18 (42%)	10 (9%)	**<.01**
Penicillin allergy, serious^a^	6 (14%)	1 (1%)	**<.01**
Charlson Comorbidity Index^b^ (IQR)	8.3 (3, 12)	7.4 (3.5, 11)	.36
Number of predisposing conditions (IQR)	2.5 (1.5, 3)	2.1 (1,3)	.08
Active solid organ malignancy	3 (7%)	15 (13%)	.40
Active hematological malignancy	3 (7%)	9 (8%)	1
History of cancer	16 (37%)	19 (17%)	**.01**
History of transplant	3 (7%)	6 (5%)	.70
Neutropenia (ANC<500)	3 (7%)	6 (5%)	.70
Liver foreign body	2 (5%)	4 (3%)	.66
Underlying GI condition^c^	5 (12%)	14 (12%)	1
Underlying GU condition^c^	7 (16%)	28 (24%)	.37
HIV	0 (0%)	2 (2%)	1
Chronic kidney disease	6 (14%)	12 (10%)	.74
End stage renal disease	10 (23%)	4 (3%)	**<.01**
Prosthetic heart valve	5 (12%)	15 (13%)	1
Chronic liver disease	3 (7%)	6 (5%)	.70
Diabetes mellitus	18 (42%)	42 (37%)	.67
Cardiac implantable electronic device	5 (12%)	13 (11%)	1
Central line	9 (21%)	20 (17%)	.78
Other preexisting condition^c^	6 (14%)	14 (12%)	.98
Microbiology			
Species			
*E. faecalis*	37 (86%)	96 (83%)	.88
*E. faecium*	4 (9%)	15 (13%)	.60
*E. avium*	1 (2%)	3 (2%)	.89
*E. casseliflavus*	1 (2%)	1 (1%)	.47
Resistance			
Penicillin-R	3 (7%)	2 (2%)	.12
Vancomycin-R	2 (5%)	4 (3%)	.66
Daptomycin-R^d^	3/41 (7%)	0/113 (0%)	**.02**
Infection			
Duration of bacteremia (day, IQR)	1.7 (1, 2)	1.8 (1, 2)	.56
Source of bacteremia			
GU	9 (21%)	30 (26%)	.64
GI	10 (23%)	32 (28%)	.71
Central line	8 (19%)	8 (7%)	.06
IVDU	2 (5%)	9 (84%)	.73
Unknown	11 (25%)	27 (23%)	.95
Other^e^	3 (7%)	9 (8%)	1
Polymicrobial	17 (40%)	18 (16%)	**<.01**
Metastatic	5 (12%)	36 (31%)	**.02**
Endocarditis	3 (7%)	27 (23%)	**.02**
Management			
Treatment duration (week) (IQR)	3.0 (2, 4)	3.4 (2, 6)	.42
Combination therapy	0 (0%)	26 (23%)	**<.01**
Echocardiogram	23 (53%)	72 (63%)	.39
ID consultation	35 (81%)	93 (81%)	1
OPAT	15 (35%)	47 (41%)	.62
Outcome			
30-day all-cause mortality	2 (5%)	9 (8%)	.73
90-day all-cause mortality	4 (9%)	22 (19%)	.16
30-day relapse	1 (2%)	2 (2%)	1
Length of stay (day, IQR)	22 (5, 32)	21 (6, 22)	.82

IQR: interquartile range; ANC: absolute neutrophil count; R: resistant; GU: genitourinary; GI: gastrointestinal; HIV: human immunodeficiency virus; IVDU: intravenous drug use; ID: infectious disease; OPAT: outpatient parenteral antimicrobial therapy

a: documented anaphylaxis or respiratory difficulty following penicillin administration; b: DenoDH score, which excludes HIV; c: See supplementary appendix for a complete list of conditions; d: Denominators are lower due to missing antimicrobial susceptibility test results for several isolates; e: Skin and soft tissue or bone infection source, intravascular source including thrombus and cardiac vegetation, and prosthetic joint infection.

In a multivariable regression model, NBL was more likely in those with: younger age (adjusted odds ratio [AOR] .95, *P* < .01), any penicillin allergy (AOR 5.87, *P* < .01), history of cancer (AOR 5.25, *P* < .01), ESRD (AOR 12.48, *P* < .001), and polymicrobial bacteremia (AOR 4.20, *P* < .01) (Table 3). VIF showed low correlation among tested variables (1.05–1.59).

**Table 3.** Factors associated with use of non-beta lactam antibiotic for definitive therapy of ampicillin-susceptible *Enterococcus* bacteremia: multivariable logistic regression

Variable	Adjusted Odds Ratio (95% CI)	95% CI	*P*-value
Age	0.95	0.92–0.98	**<.01**
Penicillin allergy, any	5.87	2.04–17.80	**<.01**
History of cancer	5.25	1.65–17.95	**<.01**
End stage renal disease	12.48	3.19–56.40	**<.001**
Endocarditis	0.26	0.05–1.04	.05–.1
Polymicrobial	4.20	1.54–11.82	**<.01**

## Discussion

We found that NBL agents were used to definitively treat ASEB in 27% of cases at our hospital from 2016–2021. This rate was higher than expected. Although not considered bactericidal when used as monotherapy against *Enterococcus*,^13^ BL agents (classically ampicillin) are the first line agents for enterococcal infections^14^ with the most historical clinical experience. We identified several factors associated with NBL use in ASEB. Some, such as penicillin allergy, are intuitive from a medical perspective. Other factors are linked not only to medical needs of patients, but also to postdischarge care coordination. For patients with ESRD on hemodialysis, vancomycin and daptomycin offer ideal postdialysis dosing at the dialysis center, obviating the need for long-term central venous access required with BL therapy - an important consideration to preserve upper extremity veins for future vascular access needs. This postdialysis antibiotic arrangement also lessens care coordination needs, as these patients do not need postdischarge home infusion services or home health nursing services. Impact of care coordination in antimicrobial prescribing pattern is a topic that warrants additional research.

Other factors we identified as associated with NBL use are: younger age, history of cancer, and polymicrobial infection. We hypothesize that younger patients may be more likely to receive NBL because they have fewer medical comorbidities. Analysis of our data set showed that younger age was statistically significantly associated with a lower number of author-defined comorbidities and CCI. It is possible that the perception of a healthier host may have affected the clinician’s choice to treat with NBL. It is unclear why a history of cancer was a factor associated with NBL therapy, and active malignancy was not; additional studies are needed to determine the significance of this finding. Patients with polymicrobial infection often have mixed source of infection such as bowel perforation, and in these cases *Enterococcus* may have been considered to be a secondary pathogen.

All patients who received combination therapy (23%) - reserved for serious infections such as endocarditis - were on concurrent BL and not NBL therapy. Patients receiving combination therapy were mostly those with endocarditis, for which ampicillin and ceftriaxone (historically aminoglycoside) are the standard of care. Although not statistically significant, 90-day all-cause mortality was higher for those receiving BL vs NBL (19% vs 9%, *P* = .16). A larger sample size could help to determine if this is significant, and possible associated factors (eg, source, invasiveness of infection).

We found relatively low rates of 30- and 90-day mortality compared to what is reported in the literature. It is important to note that we excluded patients who died or transitioned to hospice or comfort care within days of initial bacteremia, before the antimicrobial agent regimen was finalized. Our goal was to focus on determining outcomes of antimicrobial treatment. Attributing mortality due to enterococcal bacteremia remains a difficult task.

There are potential stewardship implications of using NBL for ASEB. Use of broad-spectrum NBL to treat ASEB may select for NBL-resistant enterococci. Daptomycin-nonsusceptible enterococci have been reported to emerge during prolonged treatment with daptomycin, especially in cases with a deep-seated infection.^15^ A single-institution study showed that rates of bacteremia caused by daptomycin-resistant VRE increased over time.^16^ Additional research is needed to determine whether the resistance implications of NBL use for ASEB are worth the potential benefit of easier dosing, avoidance of long-term central venous catheters, and practical discharge considerations.

Our study has several strengths and limitations. We conducted a large real-world analysis that focused on NBL use to treat ASEB in a recent era when the most frequently used NBL agents (vancomycin, daptomycin, and linezolid) were generic and widely available. We studied clinical factors that are often considered by physicians in treating patients with EB. Our study has several limitations. It is a single-center study with significant racial and ethnic homogeneity, which may impact generalizability. We did not assess antimicrobial dosing, which may be relevant in the context of changing daptomycin dosages used to treat EB. A significant proportion of included patients had unknown source of bacteremia. We did not obtain information on treatment-related adverse effects such as *C. difficile* colitis, renal, or hepatic toxicities associated with treatment antibiotics. The complexity of each individual patient and their prolonged hospital course makes that determination difficult.

We conclude that NBL can be used to treat ASEB in select patients, with good clinical outcomes. Various factors may influence the clinician decision to use NBL versus BL, including ease of arranging for long-term antimicrobials, the patient’s underlying comorbidities, and perceived severity of illness. Future research should assess the outcomes of NBL versus BL use in specific populations, for example, in patients with ESRD. Within the limitations of this study, there was not an obvious difference in clinical outcomes.

## Supporting information

10.1017/ash.2025.10078.sm001 Kang et al. supplementary material Kang et al. supplementary material

## Supplementary material

The supplementary material for this article can be found at https://doi.org/10.1017/ash.2025.10078.
