# Supplementary material for: Non-beta-lactam agents for definitive treatment of ampicillin-susceptible Enterococcus bacteremia: a single-center experience
Source: Antimicrob Steward Healthc Epidemiol. 2025 Aug 11;5(1):e182. doi: 10.1017/ash.2025.10078 (PMC12345058; doi:10.1017/ash.2025.10078)
Supplement: Kang et al. supplementary material [file S2732494X25100788sup001.docx]

**Supplementary Appendix**

**Supplementary Appendix Table 1. Author-defined preexisting conditions**

| **Liver foreign body** | Biliary obstruction, biliary stent, transjugular intrahepatic portosystemic shunt (TIPS) |
| --- | --- |
| **Underlying GI disease** | Post-operative intra-abdominal infection, bowel perforation or fistula, post-operative abnormal anatomy (e.g. anastomotic stricture following hepaticojejunostomy), radiation proctitis, irritable bowel disease, peritonitis, pancreatitis, cholangitis, cholecystitis |
| **Underlying GU disease** | Bladder or kidney stone, hydronephrosis, bladder dysfunction due to obstructive uropathy (e.g. ureteral obstruction, benign prosthetic hypertrophy), presence of chronic suprapubic catheter or percutaneous nephrostomy tube, ureteral stent, chronic foley catheter, urostomy, fistula involving bladder |
| **Other predisposing conditions** | Active alcohol use disorder, ankylosing spondylitis, anorexia with malnutrition, bicuspid aortic valve, chronic osteomyelitis, intravascular thrombus, graft-versus-host disease, hemophagocytic lymphohistiocytosis, history of endocarditis managed medically, hypogammaglobulinemia, incompletely treated enterococcal bacteremia, leukocytoclastic vasculitis, mixed connective tissue disease, prolonged hospitalization due to COVID-19, polymyalgia rheumatica, rheumatoid arthritis |

**Supplementary Appendix Table 2. Temporal trend of NBL use to treat ASEB over time**

| Calendar Year | Beta lactam | Non-beta lactam (%) | p-value^a^ |
| --- | --- | --- | --- |
| 2016 | 18 | 9 (33%) | 0.16 |
| 2017 | 17 | 8 (32%) |  |
| 2018 | 17 | 6 (26%) |  |
| 2019 | 20 | 5 (20%) |  |
| 2020 | 19 | 8 (30%) |  |
| 2021 | 24 | 7 (23%) |  |
| ^a^Chi-square for trend | | | |
